# Patient satisfaction with task shifting of antiretroviral services in Ethiopia: implications for universal health coverage

**DOI:** 10.1093/heapol/czu072

**Published:** 2014-09-11

**Authors:** Elias Asfaw, Sarah Dominis, John G H Palen, Wendy Wong, Abebe Bekele, Amha Kebede, Benjamin Johns

**Affiliations:** ^1^Ethiopian Health and Nutrition Research Institute (EHNRI), P.O. Box 1242, Addis Ababa, Ethiopia, ^2^Abt Associates Inc., 4550 Montgomery Avenue, Bethesda, MD 20814, USA and ^3^U.S. State Department, Office of Global AIDS Coordinator, 2100 Pennsylvania Ave NW, Suite 200, Washington, DC 20037

**Keywords:** Africa, AIDS, antiretroviral services, attitudes, developing countries, Ethiopia, health care utilization, health professionals, health systems research, health workforce, health workers, HIV, human resources for health, international health policy, labour market, patients, patient satisfaction, people-centred health systems, task sharing, task shifting, universal health access, universal health coverage, workforce shortages

## Abstract

Formalized task shifting structures have been used to rapidly scale up antiretroviral service delivery to underserved populations in several countries, and may be a promising mechanism for accomplishing universal health coverage. However, studies evaluating the quality of service delivery through task shifting have largely ignored the patient perspective, focusing on health outcomes and acceptability to health care providers and regulatory bodies, despite studies worldwide that have shown the significance of patient satisfaction as an indicator of quality. This study aimed to measure patient satisfaction with task shifting of antiretroviral services in hospitals and health centres in four regions of Ethiopia. This cross-sectional study used data collected from a time–motion study of patient services paired with 665 patient exit interviews in a stratified random sample of antiretroviral therapy clinics in 21 hospitals and 40 health centres in 2012. Data were analyzed using *f*-tests across provider types, and multivariate logistic regression to identify determinants of patient satisfaction. Most (528 of 665) patients were satisfied or somewhat satisfied with the services received, but patients who received services from nurses and health officers were significantly more likely to report satisfaction than those who received services from doctors [odds ratio (OR) 0.26, *P* < 0.01]. Investments in the health facility were associated with higher satisfaction (OR 1.07, *P* < 0.01), while costs to patients of over 120 birr were associated with lower satisfaction (OR 0.14, *P* < 0.05). This study showed high levels of patient satisfaction with task shifting in Ethiopia. The evidence generated by this study complements previous biomedical and health care provider/regulatory acceptability studies to support the inclusion of task shifting as a mechanism for scaling-up health services to achieve universal health coverage, particularly for underserved areas facing severe health worker shortages.

**KEY MESSAGES**

- This study brings the patient perspective to complement previous studies which evaluated task-shifting quality from the perspective of biomedical health outcomes and health care provider/regulatory body acceptability.
- Patients receiving largely routine ART services from nurses and health officers in an Ethiopian task shifting environment were at least as satisfied as those receiving services from physicians.
- Patients receiving ART services in Ethiopia from nurses through task shifting reported greater satisfaction with friendly services, information about medications, prompt attention and provision of all services needed than those receiving services from physicians or health officers.

## Introduction

In December 2012, the United Nations unanimously adopted a resolution which recognized the responsibility of governments to accelerate the transition towards providing universal coverage of affordable and quality health care services (United Nations General Assembly 2012). As with the scale up of antiretroviral therapy (ART) services in sub-Saharan Africa in the past decade, the focus on universal health coverage (UHC) has brought the global health workforce shortage to the forefront of the global health agenda (WHO 2006). Access to health services is predicated on an adequate and equitably distributed health workforce; the composition of the workforce has implications for affordability and quality (Fifty-Eight World Health Assembly 2005; WHO 2010a; Scheil-Adlung and Bonnet 2011; United Nations General Assembly 2012).

Task shifting’s success in rapidly scaling up ART services may make it a promising mechanism for accomplishing UHC. Task shifting has long been used informally in the health field. In 2008, the World Health Organization (WHO) released guidelines for formalizing task shifting of HIV services through modifying health worker scopes of practice (WHO 2008; Lehmann *et al.* 2009; Callaghan *et al.* 2010). Countries adopting task shifting as a formal policy were able to scale-up ART rapidly. For example, Malawi launched ART services using non-physician clinicians (NPCs) in 2004, and by 2007, 130 448 patients had started ART at 154 facilities (Zachariah *et al.* 2009). Ethiopia adopted task shifting as an element of decentralization and ART services increased from three facilities in 2004 to 743 in 2010 (Ethiopia National AIDS Resource Center 2012). Task shifting has proven particularly valuable for underserved populations in rural areas, where retention of physicians is a significant challenge (WHO 2010b). However, although task shifting improves availability of health services, the quality of services delivered and its relation with patient satisfaction must be considered before recommending its use for UHC.

Task shifting’s effect on health outcomes has been well studied. Both within and outside ART service delivery, task shifting to NPCs has been shown to have similar morbidity and mortality outcomes to physician-centred models (Vaz *et al.* 1999; Rowe *et al.* 2001, 2003; Wilson *et al.* 2005; Lin and Franco 2007; Shumbusho *et al.* 2009; Callaghan *et al.* 2010; Fairall *et al.* 2012; Mdege *et al.* 2012). Yet, Scheil-Adlung and Bonnet (2011) contends that quality is a function of need not only in terms of medical, but also social, dimensions. Task shifting has been shown to be acceptable to health care providers and regulatory bodies (Dovlo 2004; Mullen and Frehywot 2007; Daviaud and Chopra 2008; Medecins Sans Frontieres 2008; Zachariah *et al.* 2009). However, the social dimension of patient preference has been less explored.

Worldwide, patient satisfaction has been recognized as an important factor in measurement of quality of services and as a contributor to outcomes of care (Donabedian 1987; Davies and Ware 1988; Barbosa *et al.* 2012). Patients’ decisions to utilize services are impacted by the quality of care they believe they will receive, and some studies have shown that more satisfied patients have better adherence to medications (Mullen and Frehywot 2007; Scheil-Adlung and Bonnet 2011; Tateke *et al.* 2012).

### Research purpose

The aim of our study was to measure patient satisfaction with task shifting of ART services in hospitals and health centres in Ethiopia. Our null hypothesis is that patients would have the same level of satisfaction with services provided by health officers and nurses as with those provided by physicians. As secondary analyses, we explore the relationship between other factors measured during the patients’ visit and satisfaction, including waiting times, reason for visit, WHO stage at the visit and time spent with the provider. We also explore the relationship between facility characteristics and satisfaction.

## Materials and methods

### Study design

This is a cross-sectional study, using data collected from a stratified random sample of ART clinics in four regions of Ethiopia in 2012.

### Facility sampling

We used a stratified random sample to select health facilities for the study. Four regions of Ethiopia were selected for inclusion based on variables we considered relevant for a representative sample, such as density of doctors, ART patient load and population density. The regions—Addis Ababa, Amhara, Benishangul Gumuz and Oromiya—served as strata for further sampling. ‘Health networks’ in Ethiopia represent a system linking individual hospitals and the health centres associated with them. We sampled health networks within the selected regions with certainty if a physician was involved in ART care at the hospital and randomly sampled the health networks that did not have physicians serving in the hospital.

The selected health networks served as strata for further sampling. In each selected network, the hospital was included in the study, and we sampled to select two or three of the network’s health centres that had provided ART services for at least 2 years. We selected all health facilities in networks with three or fewer eligible health centres. In networks with four or more eligible health centres, we stratified facilities by length of ART delivery. We randomly selected one health centre from the strata with the longest history of ART services, and then randomly selected two from all remaining facilities. Finally, time–motion studies and patient exit interviews were done at the hospital and one health centre from each strata. If there were only health centres from one strata available (e.g. if all health centres had short history of ART services), we randomly sampled only one health centre for that network; these data are used for this study. See Table 1 for the final facility sample by region.

**Table 1:** Network and facility sample size

Region	Number of networks	Number of facilities
Addis Ababa	4	15
Amhara	9	32
Benishangul Gumuz^a^	2	8
Oromiya	6	24
Total	21	79

^a^One clinic was visited by data collectors despite not being part of the sample. This ‘volunteer’ clinic was given a weight of zero. Thus, total sample size is 78 facilities, with 7 from Benishangul Gumuz.

### Study subjects

The study subjects were patients accessing HIV services at sampled facilities. We also interviewed the manager at each health facility sampled.

Over the course of 1 day per facility, we obtained a consecutive sample of up to the first 15 consenting adult (>18 years) patients attending ART services meeting our inclusion criteria. Patients were excluded from the analysis if they had not yet initiated ART. We measured the time each sampled ART patient started and ended a visit with a health care provider, and providers completed a task checklist containing basic patient attributes and types of tasks done with the patient. The study did not influence which cadre of health provider patients saw—facilities followed normal practice in assigning patients to providers. We then conducted exit interviews with the sampled patients.

### Data collection

We piloted data collection tools at one hospital and one health centre. All data collectors received 3 days of training on the data collection tools before going to the field. Senior supervisors accompanied data collectors to the field and checked data quality daily.

The facility manager interview tool contained questions on ART delivery and task shifting, supervision, decentralization and number of ART patients. The tool also ascertained the number and types of staff working in the ART clinic, and how much time they spend there. A time–motion study and task checklist were used to assess staff roles, time spent with patients and services provided.

Patient exit interview tools contained questions on the patient’s ART treatment phase, reason for visit, care received, payments incurred for the visit, reason for selecting facility and satisfaction with visit. Patient satisfaction with the visit was measured using a Likert scale ranging from satisfied to dissatisfied. The tool focused exclusively on satisfaction with the services patients received on the day of the study visit. Therefore, we categorized task shifting based on the provider the patient had seen that day, which can be divided into three categories: physician, health officer and nurse. We were able to identify the type of clinician patients saw using the time–motion study, and also the type of clinician patients believed they saw through the patient exit interviews. A unique patient identification code linked the time–motion studies with patient exit interviews for results comparison.

### Statistical analysis

We compiled descriptive statistics on patient characteristics according to the type of provider seen. We used the *f*-test to determine whether at least one pair-wise comparison across the provider types showed a statistically significant difference. We defined *P* < 0.05 to be statistically significant. All analyses were done with appropriate sample weights, as the standard errors reflect. We used multivariate logistic regression with an outcome of whether the patient reported being satisfied with the visit to identify independent determinants of patient satisfaction. We ran this regression with only visit-level characteristics, and then again with both visit-level and facility-level characteristics. Note that we could not fully account for both the multi-level structure of the model and the complex survey design in the final model.

## Results

The sample where time–motion studies and patient exit interviews were conducted comprised 21 hospitals and 40 health centres. Time–motion studies, task checklists and patient exit interviews were conducted with 665 patients. Facility manager surveys were completed at each facility.

### Facility and patient characteristics

The average hospital started offering ART in 2006, while health centres started, on average, in 2008. Hospitals had an average of 1000 patients actively on ART in the year before the survey took place, while health centres had about 250. Nearly all facilities were in urban areas. All the patients seeing physicians received their care at a hospital, while 61% (*N* = 79) of those seeing a health officer and 40% (*N* = 205) of the patients seeing a nurse received care at a health centre.

Of the 665 patients sampled, 77% (*N* = 512) saw a nurse, 20% (*N* = 130) a health officer and 3% (*N* = 23) a physician. However, when we compared the provider patients had seen with the provider patients reported seeing using linked time–motion and exit interview data, we found that 35% (*N* = 232) of patients mistook their health provider as belonging to a different cadre (*P* < 0.001). Those seeing a health officer thought they had seen a nurse 10% (*N* = 13) of the time, and 20% (*N* = 36) did not know who they had seen. Those seeing a nurse thought they had seen a physician 7% (*N* = 36) of the time, but more often (26%, *N* = 134) did not know who they had seen. Patients seeing a physician were slightly less likely to be confused, but still 26% (*N* = 6) thought they had seen a nurse.

Consistent with the principles of task shifting, physicians tended to see more complicated patients. Seventy per cent (*N* = 16) of the physicians’ patients were WHO Stage III, and 72% (*N* = 17) presented for non-routine care. Approximately half of the patients seeing nurses and health officers were Stage I, with the remainder of their patients distributed among Stages II, III and IV (*P* < 0.001), respectively. Most patients seeing nurses received routine care (70%, *N* = 358), while health officers saw a mix of both routine and non-routine patients (*P* < 0.001). Patients receiving care from a physician or at a hospital incurred significantly greater out-of-pocket payments than those visiting health officers and nurses or health centres (*P* < 0.01 and *P* < 0.04, respectively).

Despite the difference in levels of complexity, patients had an average of 7.5 min of consultation per visit, regardless of what type of provider they saw (Table 2). However, patients waited longer to see physicians than other providers. Sixty-one per cent (*N* = 14) of patients seeing a doctor waited for over 1 h, as opposed to 46% (*N* = 60) and 41% (*N* = 210) seeing health officers and nurses, respectively (*P* < 0.002) (Table 2). See Table 2 for additional detail on the patient summary characteristics.

**Table 2:** Summary statistics for sampled patients

Variable	Value	Health worker seen			*P* > *f*
		Physician (*n* = 23)	Health officer (*n* = 130)	Nurse (*n* = 512)	
*Current patient visit*					
WHO stage at visit (%)	Stage 1	2 (10%)	68 (52%)	237 (46%)	<0.001**
	Stage 2	5 (19%)	40 (31%)	118 (23%)	
	Stage 3	16 (70%)	18 (14%)	147 (29%)	
	Stage 4	0 (0%)	4 (3%)	10 (2%)	

Patient’s reason for visit	Routine visit	6 (28%)	76 (58%)	357 (70%)	<0.001**
	Initiation	0 (0%)	0 (0%)	11 (2%)	
	Non-routine visit (includes OIs, laboratories, etc.)	17 (72%)	54 (41%)	144 (28%)	

Care received at visit	Routine care	5 (22%)	77 (59%)	309 (60%)	<0.001**
	Initiation	0 (0%)	0 (0%)	11 (2%)	
	Non-routine care (includes OIs, laboratories, etc.)	18 (78%)	53 (41%)	192 (37%)	

Waiting time (min)	0–30	5 (20%)	27 (21%)	112 (22%)	0.002**
	31–60	2 (10%)	29 (22%)	124 (24%)	
	61–119	8 (35%)	38 (29%)	137 (27%)	
	120 or greater	6 (26%)	23 (17%)	69 (14%)	
	Missing	2 (10%)	13 (10%)	70 (14%)	

Time with provider	Minutes (SE)	7.4 (0.5)	7.5 (1.3)	7.5 (1.3)	0.98

Costs incurred (transportation, hospital fees)	Ethiopian birr (SE)	59.1 (14.5)	7.5 (1.4)	11.5 (2.1)	0.010*

*Facility characteristics*					

Art services integrated with non-HIV services	No	21 (91%)	63 (49%)	287 (56%)	0.555
	Yes	2 (9%)	67 (51%)	225 (44%)	

Length of time facility has offered ART services	Started before EFY2000	23 (100%)	111 (86%)	409 (80%)	0.583
	Started after EFY2000	0 (0%)	19 (14%)	103 (20%)	

Doctor present at facility ART service, even if not seen	No	0 (0%)	94 (72%)	311 (61%)	0.414^a^
	Yes	23 (100%)	36 (28%)	201 (39%)	

Improvements made to facility infrastructure	No	0 (0%)	4 (3%)	47 (9%)	0.306^a^
	Yes	23 (100%)	126 (97%)	465 (91%)	

Number of ART patients in the past 12 months	Total (SE)	3922 (848)	1559 (294)	2043 (514)	0.057

Location	Urban	20 (89%)	130 (100%)	447 (87%)	<0.001**
	Rural	3 (11%)	9 (0%)	48 (9%)	
	Missing	0 (0%)	0 (0%)	17 (3%)	

Type of facility	Health centre	0 (0%)	80 (61%)	202 (40%)	0.109
	Hospital	23 (100%)	50 (39%)	310 (60%)	

OIs = Opportunistic Infections; SE = Standard Error; EFY = Ethiopian Fiscal Year.

^a^Excludes facilities where patient saw a doctor.

*Significant at *P* < 0.05.

**Significant at *P* < 0.01.

### Outcomes

In the exit survey, 528 of 665 patients reported that they were either satisfied or somewhat satisfied with the services they had received during the visit. In our analysis, we compared satisfaction with the provider patients had actually seen, rather than the provider patients believed they had seen. We found that, when considering patients responding either ‘satisfied’ or ‘somewhat satisfied’, satisfaction levels were similar across provider types. However, when considering only those selecting ‘satisfied’, the patients seeing nurses and health officers had much higher satisfaction levels (65%, *N* = 333) than patients seeing physicians (39%, *N* = 9) (*P* < 0.001) (Table 3).

**Table 3:** Patient selection of facility criteria and satisfaction with services, by provider seen

Variable	Scale	Health worker seen			*P* > *f*
		Physician (*n* = 23)	Health officer (*n* = 130)	Nurse (*n* = 512)	
*Reason for choosing facility*					

Closest to home	Strongly agree	9 (41%)	81 (62%)	268 (52%)	<0.001**
	Did not strongly agree	14 (59%)	41 (32%)	220 (43%)	
	Not applicable or missing	0 (0%)	8 (6%)	24 (5%)	

Preferred health care provider works at facility	Strongly agree	6 (27%)	52 (40%)	209 (41%)	<0.001**
	Did not strongly agree	17 (73%)	73 (56%)	266 (52%)	
	Not applicable or missing	0 (0%)	5 (4%)	37 (7%)	

Meets all health needs	Strongly agree	7 (32%)	55 (42%)	208 (41%)	<0.001**
	Did not strongly agree	16 (68%)	70 (54%)	274 (53%)	
	Not applicable or missing	0 (0%)	5 (4%)	30 (6%)	

Will not meet people known community members	Strongly agree	14 (60%)	54 (42%)	253 (49%)	<0.001**
	Did not strongly agree	9 (40%)	68 (52%)	231 (45%)	
	Not applicable or missing	0 (0%)	8 (6%)	28 (5%)	

Satisfaction with service components					

Friendly service	Satisfied	19 (82%)	109 (84%)	453 (89%)	<0.001**
	Less than satisfied	4 (18%)	13 (10%)	42 (8%)	
	Not applicable or missing	0 (0%)	8 (6%)	17 (3%)	

Information about medication provided	Satisfied	15 (64%)	111 (85%)	461 (90%)	0.014*
	Less than satisfied	6 (27%)	12 (9%)	34 (7%)	
	Not applicable or missing	2 (9%)	7 (5%)	17 (3%)	

Information about ailment provided	Satisfied	15 (64%)	113 (87%)	441 (86%)	0.056
	Less than satisfied	6 (27%)	10 (8%)	52 (10%)	
	Not applicable or missing	2 (9%)	7 (5%)	19 (4%)	

Prompt attention	Satisfied	7 (31%)	85 (65%)	344 (67%)	<0.001**
	Less than satisfied	16 (69%)	39 (30%)	146 (28%)	
	Not applicable or missing	0 (0%)	6 (4%)	22 (4%)	

Information about what cash payment was for provided	Satisfied	2 (9%)	41 (31%)	150 (29%)	0.014*
	Less than satisfied	4 (17%)	30 (23%)	92 (18%)	
	Not applicable or missing	17 (74%)	59 (46%)	270 (53%)	

All services believed needed provided	Satisfied	10 (46%)	109 (84%)	433 (85%)	<0.001**
	Less than satisfied	11 (46%)	12 (9%)	51 (10%)	
	Not applicable or missing	2 (9%)	9 (7%)	28 (5%)	

Referrals needed provided	Satisfied	9 (41%)	40 (31%)	141 (28%)	0.028*
	Less than satisfied	4 (17%)	26 (28%)	185 (36%)	
	Not applicable or missing	10 (42%)	54 (41%)	186 (36%)	

*Overall patient satisfaction*					

Patient's satisfaction	Satisfied	8 (34%)	84 (65%)	334 (65%)	<0.001**
	Somewhat satisfied	9 (39%)	18 (13%)	75 (15%)	
	Neither satisfied or dissatisfied	2 (10%)	2 (2%)	12 (2%)	
	Somewhat dissatisfied	0 (0%)	1 (1%)	6 (1%)	
	Dissatisfied	0 (0%)	2 (2%)	1 (0%)	
	Missing	4 (17%)	23 (18%)	84 (16%)	

*Significant at P < 0.05.

**Significant at P < 0.01.

To determine the predictors of patient satisfaction, we conducted bivariate and multivariate regression of provider type to patients’ overall satisfaction. In the bivariate analysis, we found that patients were significantly less likely to be satisfied receiving services from a doctor than a nurse [odds ratio (OR) 0.2, *P* < 0.01], and had similar satisfaction levels between a health officer and a nurse. This effect was comparable in the multivariate regression model (OR 0.26, *P* < 0.01), which controlled for the patients’ WHO stage, reason for visit, waiting time and time with provider, costs incurred and wages lost. Although patients seeing doctors were sicker, waited longer to see the provider and spent the same length of time (7.5 min) with doctors as patients seeing other providers, these variables were not significant predictors of satisfaction in the final multivariate regression model. Only two additional variables had a significant effect on overall patient satisfaction. Significantly higher cost (>120 birr) to patients was associated with significantly decreased satisfaction (OR 0.14, *P* < 0.05), while improvements to the facilities for ART services significantly increased satisfaction (OR 3.04, *P* < 0.01). In bivariate regression, patients’ WHO HIV stage was also significant, with Stage IV patients less satisfied than Stage I (OR 0.38, *P* < 0.01), but the patients’ stage was not significant in the final multivariate model. See Table 4 for the final model.

**Table 4:** Predictors of overall patient satisfaction with ART at facility, by provider seen (multivariate regression)

Variable (outcome = satisfied)	Comparator	Category	Odds ratio of reporting satisfaction	Linearized SE
Provider seen	Nurse	Doctor	0.26	0.09**
		Health officer	0.96	0.39
WHO stage at visit (%)	Stage 1	Stage 2	0.96	0.46
		Stage 3	1.08	0.39
		Stage 4	0.74	0.41
Patient's reason for visit/care received	Reason for visit and care received same	Not the same	0.9	0.24
Waiting time (min)	≥10	11–29	0.53	0.3
		30–59	1.37	0.9
		60–119	0.88	0.39
		120 or greater	1.07	0.61
		Missing	0.68	0.33
Time with provider (min)	≥5	>5–10	1.18	0.28
		>10–20	0.56	0.19
		>20–30	0.4	0.3
		>30	0.16	0.24
Costs incurred (transportation, hospital fees)	No cost	>0 and < 5 birr	0.89	0.31
		>5–120 birr	0.82	0.19
		>120 birr	0.14	0.12*
Wages lost for this visit	Did not lose wages	Lost wages	1.13	0.47
		Missing	1.32	0.71
*Facility characteristics*				
ART services integrated with non-HIV services	No	Yes	0.97	0.47
Length of time facility has offered ART services	Started before EFY2000	Started after EFY2000	0.79	0.21
Length of time facility has offered ART services with task shifting	Started before EFY2000	Started after EFY2000	0.46	0.19
Doctor present at facility ART service, even if not seen	No	Yes	1.19	0.79
Improvements made to facility infrastructure	No	Yes	3.04	1.07**
Number of ART patients in the past 12 months	Up to 100	>100–500	0.93	0.99
		>500–1000	0.67	0.74
		>1000–2000	0.28	0.36
		>2000	0.13	0.16
In the last year, clinical mentoring provided for Task Shifting	Yes	No	0.85	0.28
		Missing	2.45	1.07
Location	Urban	Rural	1.38	0.7
		Missing	1.68	0.62
Type of facility	Health centre	Hospital	1.1	0.86

*Significant at *P* < 0.05.

**Significant at *P* < 0.01.

In pair-wise comparisons of patient satisfaction with individual aspects of services received, we found significant differences between types of providers, with patients reporting higher satisfaction with nurses and health officers than doctors on most individual aspects. However, patients reported a higher level of satisfaction with the referrals provided by their physicians than from health officers and nurses (*P* < 0.028). Patients were more satisfied with the way nurses and health officers provided information about their medications and what their cash payment was for (*P* < 0.014 and *P* < 0.014, respectively). When asked about friendly service, satisfaction ranged from 82% (*N* = 19) for doctors to 89% (*N* = 456) for nurses (*P* < 0.001). Patients also reported higher satisfaction in receiving all the services they needed and more prompt attention from nurses and health officers than with doctors (*P* < 0.001 and *P* < 0.001, respectively). Refer to Table 3 for detailed information.

## Discussion

This study found that patients receiving largely routine services from nurses and health officers in an Ethiopian task-shifting environment were at least as satisfied as those receiving services from physicians. This is consistent with another study of ART services in Ethiopia that found that patients generally accepted nurse and health officer services for non-severe cases in 2009 (Assefa *et al.* 2012).

Only two factors proved significant to determining patient satisfaction in our final multivariate model aside from low levels of satisfaction with physicians: cost to patient and investments in infrastructure. Satisfaction was similar across most payment levels (0 to 120 birr) but dropped off precipitously for payments over 120 birr. This finding may be relevant for the design of UHC policies, although, by its nature, the study design excludes patients who may have eschewed seeking services due to concern about costs. Investment in infrastructure raised satisfaction substantially. The study did not further investigate what aspects of infrastructure investment affected the patients’ satisfaction, but we hypothesize that more comfortable waiting and examining room conditions may have played a role. In addition, infrastructure investment may serve as a proxy for other investments, such as improved availability of medical supplies.

This study found that levels of overall satisfaction with the services provided by nurses and health officers were correlated with satisfaction for prompt attention, friendly services, the way services such as medications and service fees were explained, and the perceived comprehensiveness of services provided. The significant correlations found in these aspects of provider behaviour and patients’ overall satisfaction are consistent with the literature on patient satisfaction. Although there are many drivers of patient satisfaction, interactions with clinicians, including perceived clinician competence and provider attitudes, are among the most significant (Dang *et al.* 2012; Otani *et al.* 2012; Tateke *et al.* 2012). In a study among HIV patients in the United States, the provider of services was the most significant indicator driving satisfaction (Tateke *et al.* 2012). Similarly, in a study of public and private hospital out-patient services in Ethiopia, perceived health care provider’s technical competency, welcoming approach and adequacy of consultation duration were common drivers of satisfaction (Dang *et al.* 2012).

This study found a greater proportion of patients had to wait times 1 h and longer when seeing a physician rather than the other providers. However, we did not find an association between wait times and patient satisfaction. This is in contrast to other studies which found that wait times to see a clinician affect patient satisfaction (Nabbuye-Sekandi *et al.* 2011; Ogunfowokan and Mora 2012). It should be noted that over 40% of patients seeing a nurse or a health officer had waiting times of 1 h or more, indicating that waiting times of this length are not uncommon for ART services. This study found that patient–provider encounters were ∼7.5-min long, with little variation by the type of provider. This encounter length is on par with another study in Ethiopia, which found mean consultation duration of 7.82-min in public hospitals (Tateke *et al.* 2012).

### Limitations

There are several limitations to this study. This study looks at patient satisfaction with one provider, at one point in time, for ART services. Further research is warranted to determine patients’ satisfaction with task shifting over time, and in relation to other types of health services. The high levels of acceptance of task shifting found in Ethiopia may be impacted by the degree to which task shifting has been adopted by the country. In this study, which purposively sampled health networks providing services using physicians, only 23 patients of 665 received services from a physician. It is not clear whether the widespread use of task shifting has caused patients to adapt and increase acceptance of the practice over time. The multivariate regression controlled patient complexity or illness by using WHO stage during the visit. However, WHO staging is likely not a comprehensive measure of the patients’ illness or complexity of treating the patients’ ailments. The small number of patients seeing physicians tended to have more advanced WHO stage than patients seen by other providers, which may affect our ability to compare satisfaction of patients seen by physicians with that of the more stable patients seen by NPCs. Physicians also saw a greater proportion of patients seeking non-routine care; these patients may have been more likely to have special requests that could not be met immediately, as evidenced by the higher satisfaction levels for referrals among patients seeing physicians. Such patients likely needed more than 7.5-min consultation to adequately address their issues, and required other systemic factors that are outside of physicians’ control, such as availability of drugs and tests. The high level of satisfaction reported by patients for the physicians’ referral services may provide some support for this hypothesis. Further study of task shifting satisfaction among patients with opportunistic infections, drug side effects or special needs is warranted.

Task shifting is most often considered a strategy for scale up of services to underserved rural areas. In this study, most facilities included were urban, reflective of Ethiopia’s urban-centred distribution of health facilities. Although the patients served by these facilities were likely a mix of rural and urban patients, this study may not be representative of rural patients and rural health facilities. In addition, patients mistook the provider they were seeing a significant percentage of the time. Analysis was done on the provider seen, rather than the provider patients believed they saw. Patients’ mistaken perception of the type of provider they were seeing may have affected their level of satisfaction.

## Conclusions

From this study we can conclude that, for routine ART services among stable patients in Ethiopia, nurses and health officers provide services that are at least as satisfactory to patients as those provided by physicians. Task shifting has previously been shown to be a promising strategy in studies evaluating the quality of services delivered related to health outcomes, and provider and regulatory body acceptability. This study further bolsters the global dialogue on task shifting by providing the patients’ perspective on the practice. The evidence generated by this study supports the inclusion of task shifting as a mechanism for scaling-up health services to achieve UHC, particularly for underserved areas facing severe health worker shortages. Policy makers considering task shifting as a strategy should take into consideration the investments required to ensure the mechanism can be used safely and result in high patient satisfaction levels. Pre-service education of nurses and health officers requires less resources and time than physician pre-service training, but safe task shifting to lower cadres of health workers requires increased investment in in-service training, supportive supervision and mentorship. In addition, the retention of nurses can also be difficult, particularly in rural areas. Despite these challenges, countries seeking to increase capacity for UHC may find task shifting to be an essential strategy, and one welcomed by patients.
